# Expression of high-mobility groups box-1/receptor for advanced glycation end products/osteopontin/early growth response-1 pathway in proliferative vitreoretinal epiretinal membranes

**Published:** 2011-02-17

**Authors:** Ahmed M. Abu El-Asrar, Luc Missotten, Karel Geboes

**Affiliations:** 1 Department of Ophthalmology, College of Medicine, King Saud University, Riyadh, Saudi Arabia; 2 Department of Ophthalmology, University of Leuven, Leuven, Belgium; 3 Laboratory of Histochemistry and Cytochemistry, University of Leuven, Leuven, Belgium

## Abstract

**Purpose:**

The high-mobility group box −1 (HMGB1)/receptor for advanced glycation end products (RAGE)/osteopontin (OPN)/early growth response-1 (Egr-1) pathway is involved in inflammation, angiogenesis, and fibrosis. We investigated the expression of the components of this pathway in proliferative diabetic retinopathy (PDR) and proliferative vitreoretinopathy (PVR) epiretinal membranes.

**Methods:**

Nine active and 13 inactive membranes from patients with PDR and 21 membranes from patients with PVR were studied by immunohistochemistry.

**Results:**

In PDR membranes, vascular endothelial cells expressed HMGB1, RAGE, OPN, and Egr-1 in 21, 15, 20, and 16 membranes, respectively. Stromal cells expressed HMGB1, RAGE, OPN, and Egr-1 in 21, 20, 20, and 16 membranes, respectively. Significant correlations were detected between the number of blood vessels expressing the panendothelial cell marker CD34 and the number of blood vessels and stromal cells expressing HMGB1, RAGE, and OPN. The numbers of blood vessels and stromal cells expressing CD34, HMGB1, RAGE, and OPN and stromal cells expressing Egr-1 were significantly higher in active membranes than in inactive membranes. In PVR membranes, spindle-shaped myofibroblasts expressing α-smooth muscle actin coexpressed HMGB1, RAGE, OPN, and Egr-1.

**Conclusions:**

The HMGB1/RAGE/OPN/Egr-1 pathway may be involved in inflammatory, angiogenic and fibrotic responses in proliferative vitreoretinal disorders.

## Introduction

Proliferative diabetic retinopathy (PDR) is a serious complication of diabetes mellitus and is characterized by preretinal neovascularization and the development of fibrovascular membranes at the vitreoretinal interface. Formation of fibrovascular tissue often leads to severe visual loss due to vitreous hemorrhage and/or tractional retinal detachment. Proliferative vitreoretinopathy (PVR) is a process of fibrocellular proliferation on either side of the retina that may complicate rhegmatogenous retinal detachment. The formation and gradual contraction of these membranes causes a marked distortion of the retinal architecture and results in complex retinal detachments that are difficult to repair.

The pathogenesis of epiretinal membranes is still not well understood, but inflammatory and immunologic processes are known to be implicated [1-3]. In addition, strong evidence indicates that chronic low-grade inflammation is implicated in the pathogenesis of diabetic retinopathy. Diabetic retinal vascular leakage, capillary hypoperfusion, and endothelial cell damage are associated with leukocyte recruitment and adhesion to the retinal vasculature, findings that correlate with the increased expression of the retinal intercellular adhesion molecule-1 (ICAM-1) and the leukocyte integrin CD18 [4].

High-mobility group box −1 protein (HMGB1) or amphoterin is a nonhistone DNA-binding nuclear protein that is highly conserved during evolution and is present in most eukaryotic cells, where it stabilizes nucleosome formation and facilitates transcription. Necrotic cell death can result in passive leakage of HMGB1 from the cell, as the protein is then no longer bound to DNA. In addition, HMGB1 can be actively secreted by different cell types, including activated monocytes and macrophages, mature dendritic cells, natural killer cells, and endothelial cells. Extracellular HMGB1 functions as a proinflammatory cytokine. In addition to advanced glycation end products in diabetes, HMGB1 signals through the receptor for advanced glycation end products (RAGE), a member of the immunoglobulin superfamily of receptors, leading to activation of the transcription factor nuclear factor kappa B (NF-κB) and inducing the expression of various leukocyte adhesion molecules and proinflammatory cytokines and chemokines [5-8]. Several studies demonstrated that the HMGB1/RAGE signaling axis is involved in angiogenic [9-12] and fibrotic [13-16] disorders.

Osteopontin (OPN) is a phosphorylated acidic arginine-glycine-aspartate (RGD)-containing glycoprotein that exists both as an immobilized extracellular matrix component and as a soluble, multifunctional, proinflammatory cytokine that plays important roles in promoting inflammation [17,18], tissue remodeling, fibrosis [17,19-21], and angiogenesis [22-24]. Many of these effects are mediated by the binding of OPN to CD44 receptors and the surface integrin receptor α_v_β_3_ [22]. A recent study demonstrated that OPN is required for HMGB1 expression by fibroblasts in response to the profibrotic cytokine transforming growth factor-β (TGF-β1) [21].

Early growth response-1 (Egr-1) transcription factor, an immediate early gene, belongs to the family of zinc finger DNA-binding proteins. Normally, Egr-1 shows only a low level in tissue at baseline, but is rapidly and transiently induced by many stimuli, including hypoxia, shear stress, injury, growth factors, and cytokines. Egr-1 plays a key role in orchestrating tissue response to acute injury by activating the transcription of many proliferation-associated genes, such as platelet-derived growth factor A and B, connective tissue growth factor, vascular endothelial growth factor (VEGF), TGF-β, tumor necrosis factor-α (TNF-α), ICAM-1, fibroblast growth factor-2, insulin-like growth factor-2, interleukin-2 (IL-2) and Egr-1 itself [23,24]. Several studies demonstrated that Egr-1 has a role in the induction and progression of fibrosis [25-27] and angiogenesis [28].

In addition to NF-κB, which plays a central role in the proinflammatory signaling stimulated by RAGE ligands, recent studies have uncovered the novel finding that upregulation of Egr-1 in hypoxic endothelial cells is dependent on RAGE signaling [29]. In addition, HMGB1 enhanced Egr-1 expression and NF-κB nuclear translocation in vascular endothelial cells. Anti-RAGE antibody significantly reduced HMGB1-induced expression of Egr-1 [30]. In addition, Liu et al. [31] demonstrated that Egr-1 and OPN positively regulate expression of each other in a positive feedback loop.

Given the key roles of the HMGB1/RAGE/OPN/Egr-1 signaling axis in inflammation, angiogenesis, and fibrosis, we hypothesized that this pathway may be involved in the pathogenesis of proliferative vitreoretinal disorders. To test this hypothesis, we investigated the expression of HMGB1, RAGE, OPN, and Egr-1 in the epiretinal membranes from patients with PDR and PVR. The level of vascularization in PDR epiretinal membranes was determined by immunodetection of the panendothelial cell marker CD34.

## Methods

### Epiretinal membrane specimens

Epiretinal fibrovascular membranes were obtained from 22 patients with PDR during pars plana vitrectomy for the repair of traction retinal detachment or combined traction/rhegmatogenous retinal detachment. Using the operating microscope, the clinical ocular findings were graded at the time of vitrectomy for the presence or absence of visible new vessels on the retina or optic disc. Patients with active PDR were graded as such on the basis of visible new vessels on the retina or optic disc. Their absence indicated involuted (inactive) PDR. Active PDR was present in nine patients and inactive PDR was present in 13 patients. In addition, epiretinal membranes were obtained from 21 eyes undergoing vitreoretinal surgery for the treatment of retinal detachment complicated by PVR. Membranes were fixed in 10% formalin solution and embedded in paraffin. The study was conducted according to the tenets of the Declaration of Helsinki, and informed consent was obtained from all patients. The study was approved by the Research Centre, College of Medicine, King Saud University.

### Immunohistochemical staining

Endogenous peroxidase was abolished with 2% hydrogen peroxide in methanol for 20 min, and nonspecific background staining was blocked by incubating the sections for 5 min in normal swine serum. For OPN and RAGE detection, antigen retrieval was performed by boiling the sections in 10 mM Tris-EDTA (EDTA) buffer (pH 9; Sigma-Aldrich, Bornem, Belgium) for 30 min. For CD34, α-smooth muscle actin (α-SMA), HMGB1, and Egr-1 detection, antigen retrieval was performed by boiling the sections in 10 mM citrate buffer (pH 6) for 30 min. Subsequently, the sections were incubated with the monoclonal and polyclonal antibodies listed in Table 1. The specificity of the antibodies used is indicated in Table 2. The optimal working concentration and incubation time for the antibodies were determined earlier in pilot experiments. For RAGE, a second step was introduced using 1/20 rabbit antigoat-peroxidase (Dako, Glostrup, Denmark) mixed with 1/10 normal human serum. The sections were then incubated for 30 min with immunoglobulin conjugated to peroxidase-labeled dextran polymer (EnVision Flex; Dako, Carpinteria, CA). The reaction product was visualized by incubation for 10 min in 0.05 M acetate buffer at pH 4.9, containing 0.05% 3-amino-9-ethylcarbazole (Sigma-Aldrich) and 0.01% hydrogen peroxide, resulting in bright-red immunoreactive sites. The slides were then faintly counterstained with Harris hematoxylin (Sigma-Aldrich).

**Table 1:** Monoclonal and polyclonal antibodies used in this study.

**Primary antibody**	**Dilution**	**Incubation time**	Source*
Anti-CD34 (Clone My10) (mc)	1/50	60 min	BD Biosciences
Anti- a-Smooth muscle actin (Clone 1A4) (mc)	1/200	60 min	Dako
Anti-HMGB1 (Catalogue No. ab77302) (mc)	1/20	60 min	Abcam
Anti-RAGE (Catalogue No. ab20558) (pc)	1/100	60 min	Abcam
Anti-Osteopontin (Catalogue No. ab8448) (pc)	1/500	60 min	Abcam
Anti-Egr-1 (Catalogue No. AHP1939) (pc)	1/50	60 min	AbD Serotec

*Location of manufacturers: BD Biosciences, San Jose, CA; Dako, Glostrup, Denmark; Abcam, Cambridge, UK; AbD Serotec, Oxford, UK mc=monoclonal ; pc=polyclonal HMGB1=high-mobility group box −1; RAGE=receptor for advanced glycation end products; Egr-1=early growth response-1.

**Table 2:** Specificity of the Antibodies used in the study.

**Primary antibody**	**Specificity**
Anti-CD34	Reactive with the CD34 antigen
Anti- α-Smooth muscle actin	Labels a-Smooth muscle actin
Anti-HMGB1	Reacts with the full length human HMGB1 protein
Anti-RAGE	Recognizes amino acid sequence 385–399 of human RAGE
Anti-Osteopontin	Recognizes the full length osteopontin protein, as well as the C-terminal fragments of both thrombin and matrix metalloproteinase (MMP)-cleaved osteopontin. This antibody recognizes the 32 kDa MMP-cleaved fragment, but not the 40 kDa N-terminal fragment.
Anti-Egr-1	Recognizes human EGR1, a 57.5 kDa nuclear protein belonging to the EGR family of C2H2-type zinc-finger proteins.

HMGB1=high-mobility group box −1; RAGE=receptor for advanced glycation end products; Egr-1=early growth response-1

Omission or substitution of the primary antibody with an irrelevant antibody of the same species and staining with chromogen alone were used as negative controls. Sections from patients with colorectal carcinoma were used as positive controls. The sections from the control patients were obtained from patients treated at the University Hospital, University of Leuven, Belgium, in full compliance with the tenets of the Declaration of Helsinki.

### Quantitation

Immunoreactive blood vessels and cells were counted in five representative fields, using an eyepiece-calibrated grid in combination with the 40× objective. These representative fields were selected based on the presence of immunoreactive blood vessels and cells. With this magnification and calibration, the cells present in an area of 0.33×0.22 mm were counted. Data were expressed as mean values±standard deviation (SD) and analyzed by the Mann–Whitney test. Pearson correlation coefficients were computed to investigate the linear relationship between the variables investigated. A p value less than 0.05 indicated statistical significance. BMDP 2007 Statistical Package (BMDP Statistical Software, Los Angeles, CA) was used for the statistical analysis.

## Results

### Immunohistochemical analysis

#### Proliferative diabetic retinopathy membranes

**Figure 1:**
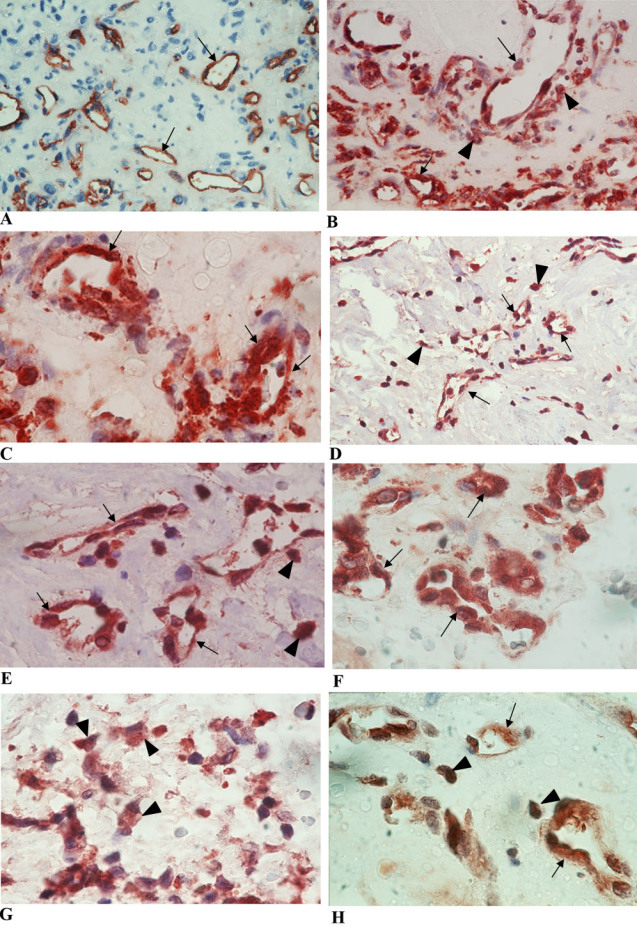
Proliferative diabetic retinopathy epiretinal membranes. **A**: Immunohistochemical staining of panendothelial cell marker CD34 shows blood vessels positive for CD34 (arrows; original magnification 40×). Immunohistochemical staining of high-mobility group box −1 (HMGB1). Low power (**B**; original magnification 40×) and high power (**C**; original magnification 100×) showing vascular endothelial (arrows) and stromal (arrowheads) cells expressing strong immunoreactivity to HMGB1. Immunohistochemical staining of receptor for advanced glycation end products (RAGE). Low-power (**D**; original magnification 40×) and high-power (**E**; original magnification 100×) showing vascular endothelial (arrows) and stromal (arrowheads) cells expressing strong immunoreactivity to RAGE. Immunohistochemical staining of osteopontin (OPN) showing vascular endothelial cells (arrows; **F**) and stromal cells (arrowheads; **G**) expressing strong immunoreactivity to OPN (original magnification 100×). Immunohistochemical staining of early growth response-1 showing immunoreactivity in vascular endothelial (arrows) and stromal (arrowheads) cells (**H**; original magnification 100×).

No staining was observed in the negative control slides. All membranes showed blood vessels positive for the panendothelial cell marker CD34 (Figure 1A), with a mean number of 29.4±30.7 (range, 1–140). Immunoreactivity for HMGB1 was present in 21 (95.5%) membranes. Strong immunoreactivity for HMGB1 was noted in the nucleus and cytoplasm of vascular endothelial cells and stromal cells (Figure 1B,C). The number of immunoreactive blood vessels ranged from 0 to 82, with a mean number of 18.3±18.7. The number of immunoreactive stromal cells ranged from 0 to 84, with a mean number of 37.9±22.0. RAGE immunoreactivity was present in 20 (91%) membranes. Strong cytoplasmic immunoreactivity for RAGE was noted in vascular endothelial cells (Figure 1D,E) in 15 (68%) membranes, with a mean number of 19.6±14.7 (range, 0–60). Strong cytoplasmic RAGE immunoreactivity was also noted in stromal cells (Figure 1D,E) in 20 (91%) membranes, with a mean number of 40.2±30.7 (range, 0–80). In serial sections, the distribution of blood vessels expressing HMGB1 was similar to the distribution of blood vessels expressing RAGE. Strong cytoplasmic immunoreactivity for OPN was present in 20 (91%) specimens. OPN immunoreactivity was noted in vascular endothelial cells (Figure 1F), with a mean number of 12.5±10.9 (range, 0–30). OPN immunoreactivity was also noted in stromal cells (Figure 1G), with a mean number of 24.4±22.9 (range, 0–75). Immunoreactivity for Egr-1 was noted in 16 (72.7%) membranes. Immunoreactivity for Egr-1 was noted in the nucleus and cytoplasm of vascular endothelial cells and stromal cells (Figure 1H). The number of immunoreactive blood vessels ranged from 0 to 30, with a mean number of 8.7±7.4. The number of immunoreactive stromal cells ranged from 0 to 60, with a mean number of 21.3±16.8.

#### Proliferative vitreoretinopathy membranes

**Figure 2:**
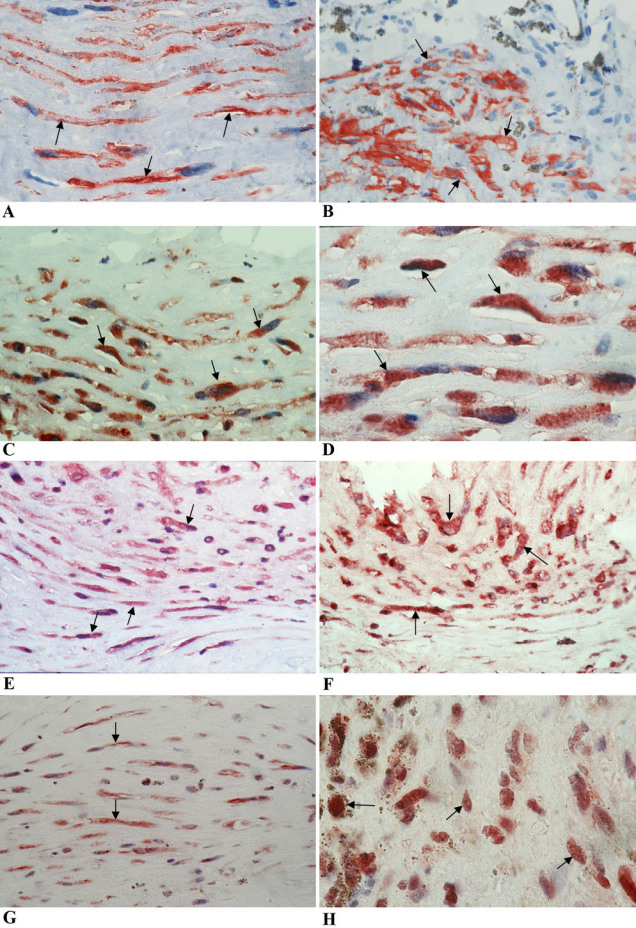
Proliferative vitreoretinopathy epiretinal membranes. Immunohistochemical staining of α-smooth muscle actin showing immunoreactivity in spindle-shaped myofibroblasts (arrows; **A**) and (**B**; original magnification 100×). Immunohistochemical staining of high-mobility group box −1 (HMGB1). Low-power (**C**; original magnification 40×) and high-power (**D**; original magnification 100×) showing spindle-shaped cells expressing cytoplasmic immunoreactivity to HMGB1 (arrows). Immunohistochemical staining of the receptor for advanced glycation end products (RAGE) showing spindle-shaped cells expressing cytoplasmic immunoreactivity to RAGE (arrows; **E**; original magnification 40×). Immunohistochemical staining of osteopontin showing strong cytoplasmic immunoreactivity in spindle-shaped cells (arrows; **F**; original magnification 40×). Immunohistochemical staining of early growth response-1 showing spindle-shaped cells expressing cytoplasmic immunoreactivity (arrows; **G**; original magnification 40×) and cells expressing nuclear immunoreactivity (arrows; **H**; original magnification 100×).

No straining was observed in the negative control slides. All membranes showed spindle-shaped myofibroblasts, expressing a strong cytoplasmic immunoreactivity for α-SMA (Figure 2A,B), with a mean number of 44.6±28.2 (range, 8–120). Immunostaining for HMGB1 revealed spindle-shaped cells expressing cytoplasmic immunoreactivity (Figure 2C,D) in 20 (95.2%) membranes, with a mean number of 40.9±24.7 (range, 0–90). Spindle-shaped cells expressing cytoplasmic immunoreactivity for RAGE (Figure 2E) were noted in 12 (85.7%) of the 14 membranes stained for RAGE, with a mean number of 55±29.1 (range, 0–120). Immunostaining for OPN revealed spindle-shaped cells expressing cytoplasmic immunoreactivity (Figure 2F) in all membranes, with a mean number of 21.2±16.5 (range, 8–70). Immunoreactivity for Egr-1 was noted in the nucleus and cytoplasm of spindle-shaped cells (Figure 2G,H) in 16 (94.1%) of the 17 membranes stained for Egr-1, with a mean number of 23.1±18.7 (range, 0–80). In serial sections, the distribution of myofibroblasts expressing α-SMA was similar to the distribution of cells expressing HMGB1, RAGE, OPN, and Egr-1.

### Correlations and relationship with proliferative diabetic retinopathy activity

The mean number of blood vessels expressing CD34, HMGB1, RAGE, and OPN, and stromal cells expressing HMGB1, RAGE, OPN, and Egr-1 was significantly higher in membranes from patients with active PDR than in membranes from patients with inactive PDR (Table 3).  Table 4 shows the Pearson correlation coefficients between the numbers of the studied variables.

**Table 3:** Mean numbers in relation to type of proliferative diabetic retinopathy (PDR).

**Variable**	**Active PDR (n=9; Mean±SD)**	**Inactive PDR (n=13; Mean±SD)**	**p-value (Mann–Whitney test)**
Blood vessels expressing CD34	54.1±35.0	12.2±7.0	<0.001*
Blood vessels expressing HMGB1	29.2±23.6	9.3±5.0	0.009*
Cells expressing HMGB1	49.0±19.7	29.5±20.5	0.046*
Blood vessels expressing RAGE	26.8±15.1	8.3±2.4	0.011*
Cells expressing RAGE	63.0±30.6	21.5±13.6	0.003*
Blood vessels expressing OPN	20.6±11.4	5.9±3.9	0.004*
Cells expressing OPN	43.0±21.1	9.2±8.4	<0.001*
Blood vessels expressing Egr-1	10.9±8.3	4.8±3.3	0.081
Cells expressing Egr-1	28.9±16.4	11.4±12.1	0.028*

*Statistically significant at 5% level of significance. Abbreviations: HMGB1=high-mobility group box −1; RAGE=receptor for advanced glycation end products; OPN=osteopontin; Egr-1=early growth response-1.

**Table 4:** Pearson correlation coefficients

**Variable**	**Blood vessels expressing CD34**	**Blood vessels expressing HMGB1**	**Cells expressing HMGB1**	**Blood vessels expressing RAGE**	**Cells expressing RAGE**	**Blood vessels expressing OPN**	**Cells expressing OPN**	**Blood vessels expressing Egr-1**
Blood vessels expressing HMGB1 r	0.946							
p	<0.001*							
Cells expressing HMGB1 r	0.625	0.621						
p	0.002*	0.002*						
Blood vessels expressing RAGE r	0.969	0.951	0.602					
p	<0.001*	<0.001*	0.023*					
Cells expressing RAGE r	0.478	0.41	0.576	0.332				
p	0.033*	0.091	0.010*	0.226				
Blood vessels expressing OPN r	0.722	0.655	0.496	0.73	0.266			
p	<0.001*	0.002*	0.031*	0.003*	0.286			
Cells expressing OPN r	0.756	0.687	0.845	0.769	0.647	0.675		
p	<0.001*	0.001*	<0.001*	0.001*	0.004*	0.004		
Blood vessels expressing Egr-1 r	0.524	0.683	0.654	0.603	0.274	0.394	0.382	
p	0.055	0.010*	0.015*	0.029*	0.344	0.163	0.177	
Cells expressing Egr-1 r	0.524	0.213	0.541	0.075	0.499	0.056	0.44	0.291
p	0.349	0.446	0.037*	0.808	0.049*	0.838	0.088	0.312

*Statistically significant at 5% level of significance. Where the row and column meet is the correlation coefficient (r) and the p-value (p) for the two variables.HMGB1=high-mobility group box −1; RAGE=receptor for advanced glycation end products; OPN=osteopontin; Egr-1=early growth response-1.

## Discussion

To gain a better understanding of the cellular and molecular processes underlying the pathogenesis of proliferative vitreoretinal disorders, we examined the expression of HMGB1, RAGE, OPN, and Egr-1 in epiretinal membranes from patients with PDR and PVR. The following four findings were demonstrated: (1) Vascular endothelial cells and stromal cells in PDR epiretinal membranes expressed HMGB1, RAGE, OPN, and Egr-1. (2) Significant correlations were observed between the number of blood vessels expressing the panendothelial cell marker CD34 in PDR epiretinal membranes and the number of blood vessels and stromal cells expressing HMGB1, RAGE, and OPN. (3) The number of blood vessels expressing CD34, HMGB1, RAGE, and OPN and the number of stromal cells expressing HMGB1, RAGE, OPN, and Egr-1 in membranes from patients with active PDR were significantly higher than that in membranes from patients with inactive PDR. (4) HMGB1, RAGE, OPN, and Egr-1 proteins were specifically localized in myofibroblasts in PVR epiretinal membranes. Taken together, our findings suggest a role for the HMGB1/RAGE/OPN/Egr-1 signaling axis in the inflammatory, angiogenic and fibrotic responses in proliferative vitreoretinal disorders via autocrine and/or paracrine mechanisms, resulting in a positive feedback loop that may contribute to the progression of PDR and PVR.

Sustained proinflammatory responses in diabetic retinopathy are often associated with angiogenesis [2-4]. The causal relationship between inflammation and angiogenesis is now widely accepted [5]. An emerging issue in diabetic retinopathy research is the focus on the mechanistic link between chronic, low-grade inflammation and angiogenesis. Recently, HMGB1 has been identified as a novel proinflammatory cytokine [5-8] that exhibits angiogenic [9-12] and fibrogenic [13-16] effects. Several studies demonstrated that RAGE mediates the inflammatory [6-8], angiogenic [9,32], and fibrogenic [15] activities of HMGB1. Another interesting role of HMGB1 in neovascularization is its ability to attract endothelial progenitor cells to sites of tissue injury and tumors to improve neovascularization in a RAGE-dependent manner [11]. HMGB1 binding to RAGE activates key cell-signaling pathways such as mitogen-activated protein kinases and NF-κB [5-8].

In the present study, HMGB1 and RAGE were colocalized in vascular endothelial cells and stromal cells in PDR epiretinal membranes. Increased vascular HMGB1 expression was demonstrated in diabetic animals [33], and recently it was demonstrated that hyperglycemia-induced reactive oxygen species production increases the expression of HMGB1 and RAGE in endothelial cells [34]. In addition, stimulation of human endothelial cells by TNF-α induced relocation of HMGB1 from the nucleus to the cytoplasm and the subsequent release of HMGB1 [35]. Activation of the HMGB1/RAGE signaling axis is important in promoting proinflammatory pathways which are considered to play an important role in diabetes-induced retinal vascular inflammation. HMGB1 activates human endothelial cells to upregulate the expression of RAGE and adhesion molecules, such as ICAM-1, vascular cell adhesion molecule-1, and E-selectin, and to release TNF-α, granulocyte colony-stimulating factor, IL-8, and monocyte chemotactic protein-1, and to increase neutrophil adhesion. This proinflammatory phenotype was mediated by the activation of NF-κB, and it was RAGE-dependent, since it was inhibited by antibodies directed toward RAGE [6-8]. In agreement with in vitro studies, systemic administration of the soluble form of RAGE inhibits blood-retinal barrier breakdown, leukostasis, and the expression of ICAM-1 in the retinas of diabetic animals [36]. RAGE also represents an important mediator of vascular oxidative stress in diabetes due to RAGE-dependent increased expression of NADPH oxidase subunits [33]. Therefore, recently, RAGE has been implicated in the pathogenesis of various diabetic complications [37].

The key cellular mediator of fibrosis is the myofibroblast, a cell type different from quiescent fibroblasts. These are contractile cells, characterized by the expression of α-SMA, and their presence is a marker of progressive disease. They have the capacity to produce several extracellular matrix components, including collagen, resulting in fibrosis [38]. In the present study, we demonstrated that strong immunoreactivities for HMGB1 and RAGE were specifically localized in α-SMA-positive myofibroblasts in PVR epiretinal membranes. This is in agreement with studies showing overexpression of HMGB1 and RAGE in other fibrotic disorders such as systemic sclerosis fibrotic skin lesions [14], idiopathic pulmonary fibrosis [13], and bleomycin-induced lung fibrosis [16]. It is also in agreement with studies showing that increased HMGB1 and RAGE expression was detected in fibroblasts in fibrotic skin lesions from patients with systemic sclerosis [14]. In a previous study, Pachydaki et al. [39] reported expression of RAGE and HMGB1 in epiretinal membranes; however, they interpreted the cells that expressed RAGE and HMGB1 to be glial cells. In vitro studies demonstrated that HMGB1 stimulated the proliferation and migration of fibroblasts through the activation of RAGE [13,15]. In addition, exposure of epithelial cells to HMGB1 resulted in the transition from an epithelial to myofibroblast-like phenotype, with a significant increase in the mesenchymal markers α-SMA and vimentin [16]. Recently, Arimura et al. [40] demonstrated that HMGB1 stimulated the migration of human retinal pigment epithelial cells, suggesting that HMGB1 may serve to promote the formation of PVR.

Several studies reported that the proinflammatory cytokine OPN plays a role in the development of diabetic vascular complications. Retinal endothelial cells under high-glucose conditions expressed increased levels of OPN and α_v_β_3_ integrin [41]. OPN expression is enhanced in human diabetic arteries [41] and in arteries of animal models of diabetes [42]. Increased renal OPN expression was also reported in human diabetic kidneys [43] and in experimental diabetic nephropathy [17]. Increased OPN expression in tubular epithelial cells of the cortex is implicated in interstitial fibrosis in experimental diabetic nephropathy [17]. In addition, vitreous OPN levels are increased in patients with diabetic retinopathy [44]. In vitro and in vivo studies demonstrated that OPN is an important angiogenic factor [45-47]. OPN also increased VEGF expression in endothelial cells [45]. Anti-α_v_β_3_ abrogated the effects of OPN on human endothelial cells [45,46]. In the present study, OPN was localized in vascular endothelial cells and stromal cells in PDR epiretinal membranes. In a previous study, we demonstrated that α_v_β_3_ integrin was expressed in PDR epiretinal membranes and was specifically localized in vascular endothelial cells and stromal cells [48]. These findings suggest that OPN can bind to α_v_β_3_ integrin in PDR epiretinal membranes and enhance angiogenesis.

OPN is required for the activation, migration, proliferation, and differentiation of fibroblasts into α-SMA-expressing myofibroblasts [19-21]. In addition, OPN is upregulated in several fibrotic diseases [17,19,20]. In the present study, OPN was specifically localized in myofibroblasts in PVR epiretinal membranes. In a previous report, we demonstrated that CD44, a cell-surface matrix adhesion receptor for OPN, was highly expressed on myofibroblast in PVR epiretinal membranes [49], suggesting that these myofibroblasts are primed to bind to OPN.

In the present study, we confirmed that Egr-1 was localized in vascular endothelial cells and stromal cells in PDR epiretinal membranes. Previous reports demonstrated that Egr-1 is activated in endothelial cells by many proangiogenic growth factors, such as VEGF and angiopoietin-1 [28]. Egr-1 was found to play an important role in angiopoietin-1-induced migration, proliferation, and the capillary-like tube formation of endothelial cells [28]. In addition, insulin or glucose upregulated Egr-1 in vascular endothelial cells, suggesting that Egr-1 may play a role in the development of vascular complications from diabetes [50].

Several studies demonstrated that the transcription factor Egr-1 has a functional role in the induction and progression of fibrosis. Bleomycin-induced scleroderma in the mouse was accompanied by increased Egr-1 nuclear expression in lesional fibroblasts [25]. In this animal model, development of fibrosis in the skin and lungs was markedly attenuated in Egr-1 null mice. Furthermore, skin-wound healing was impaired, and skin fibroblasts lacking Egr-1 showed reduced migration and myofibroblast transdifferentiation in vitro [26]. Egr-1 was also found to be upregulated by TGF-β [25] and insulin-like growth factor binding protein-5 [27] in fibroblasts, and to mediate the stimulation of extracellular matrix components transcription and fibrosis elicited by these growth factors [29,30]. In addition, Egr-1 levels were increased in vivo in lung tissues of patients with idiopathic pulmonary fibrosis [27] and in lesional skin and lung from patients with scleroderma [25]. Increased expression of Egr-1 in these fibrotic disorders was localized to fibroblasts [25,27]. Consistent with these studies, we demonstrated that Egr-1 was expressed in myofibroblasts in PVR epiretinal membranes, suggesting that Egr-1 might be one of the mechanisms responsible for the maintenance of remodeling and pathological fibrosis in this fibrotic disorder.

It should be mentioned, however, that this study inevitably has some inherent limitations, because it is based on only immunohistochemical detection of these molecules in epiretinal membranes. Several investigations to look at the level of these molecules in vitreous samples from diabetic patients will be required. However, our findings suggest that the HMGB1/RAGE/OPN/Egr-1 signaling pathway is involved in mediating inflammation, angiogenesis, and fibrosis in proliferative vitreoretinal disorders. This pathway may be a novel therapeutic target to inhibit progression of PDR and PVR.
